# Effects of botulinum toxin type A in patients with painful temporomandibular joint disorders: a systematic review and meta-analysis

**DOI:** 10.1097/MS9.0000000000002183

**Published:** 2024-05-20

**Authors:** Mengjiao Zhu, Ziwei Huang, Yeye Wang, Jing Qin, Mingyue Fan

**Affiliations:** a Department of Orthodontics, Shanghai Xuhui District Dental Center; b Department of Medical Technology, Shanghai University of Medicine and Health Sciences,Shanghai, Shanghai; c Department of Orthodontics, Nanjing Stomatological Hospital, Medical School of Nanjing University, Nanjing, People’s Republic of China

**Keywords:** botulinum toxin type A, meta-analysis, myofascial pain, temporomandibular disorders

## Abstract

**Objective:**

To assess the therapeutic efficacy of botulinum toxin type A (BTX-A) for managing myofascial pain related to temporomandibular disorders (TMDs).

**Methods:**

This study was conducted according to the PRISMA 2020 statement guidelines. The PubMed, Embase, and Cochrane Library databases were searched. Only randomized controlled trials were included. The primary outcome was a pain score on the visual analog scale, and the secondary outcomes were maximum mouth opening and adverse effects. The Cochrane risk of bias tool was used to assess risk bias. A meta-analysis of studies with the same interventions, controls, assessment methods, and follow-up durations was performed.

**Results:**

A total of 519 studies were retrieved, of which 20 randomized controlled trials were included in the qualitative analysis and six were included in the meta-analysis. The results showed that, compared with placebo, BTX-A injection was more effective at relieving myofascial pain, and its effect was similar to that of conventional methods. However, there was no difference in maximum mouth opening between the two groups. After the study assessment with the RoB 2.0 tool, six studies showed a low risk of bias, 13 studies showed some concerns regarding the reported results, and only one study showed a high risk of bias. Adverse effects of BTX-A injection were observed in four studies.

**Conclusions:**

In conclusion, BTX-A is effective at relieving pain in TMD patients but does not improve mouth opening. To minimize adverse effects, we recommend a low dose of BTX-A for TMD patients who do not experience complete pain relief from conservative treatments.

## Introduction

Highlights

- Assess the therapeutic efficacy of botulinum toxin type A (BTX-A) for managing myofascial pain related to temporomandibular disorders (TMDs).
- Provide guidance on BTX-A use in painful TMDs treatment.
- Provide a direction for future clinical studies to assess the effect of BTX-A injection in painful TMDs.

The temporomandibular joint (TMJ), composed of the temporal fossa, mandibular condyle, and a fibrocartilage disc with upper and lower cavities^1^, is a hinge type of synovial articulation that plays a critical role in coordinating daily functions such as chewing and phonation^2^. It connects the jawbone to the skull, working as a sliding hinge^3^. Many pathologies can impact the intricacies of the joint anatomy and cause clinical dysfunction, potentially leading to TMDs^4^. TMDs are commonly classified into a variety of categories, including myofascial pain, internal derangements, degenerative joint disease, chronic recurrent dislocation, and ankylosis^5^. TMDs accompanied by myofascial pain cause significant disruptions in an individual’s psychosocial functioning and significantly alter their quality of life^6^.

Numerous treatment approaches, including conservative therapies, namely, oral appliances, drugs (such as anti-inflammatory agents and muscle relaxants), warm compresses, low-level laser therapy, and behavioral therapy, have been proposed for managing pain in TMD patients. More invasive procedures, such as dry needling and acupuncture, can also treat this condition. However, for persistent symptoms such as intense and frequent pain, failure of conservative therapy is common. BTX-A has recently been used as an alternative option for treating chronic refractory myofascial pain^7^.

BTX-A is an exotoxin synthesized by the gram-positive anaerobic bacterium *Clostridium botulinum* ^8^. It induces muscle contraction relaxation and inflammatory pain relief by not only inhibiting acetylcholine exocytosis from nerve end plates but also blocking the release of substance P and glutamate^9^. BTX-A is a well-known treatment used to reduce the appearance of facial wrinkles, but it is also used for treating headaches, neuropathic facial pain, and facial nerve palsy^10^. Due to its muscle relaxing and pain-relieving effects, its clinical use has expanded to the management of musculoskeletal pain disorders, including TMD-related myofascial pain. Recently, an increasing number of studies have suggested the potential therapeutic role of BTX-A in the management of myofascial pain associated with TMD^9,11^. A previous review published in 2019 reported that the therapeutic efficacy of BTX-A was unclear^12^. More recently, based on published research data from 2008 to 2020, Ramos-Herrada *et al.* ^7^. concluded that BTX is as effective as conventional treatments for controlling myofascial pain related to TMDs and might be a useful clinical alternative to existing conservative treatments for refractory myofascial pain related to TMDs. However, the studies included in this systematic review were limited, and the evidence was of medium to low certainty. Therefore, the efficacy of BTX-A treatment is still unclear. Furthermore, BTX-A for treating TMD patients has not been approved by the FDA, possibly due to a lack of adequate research^13^. Since numerous new randomized controlled trials (RCTs) have been published in the past 3 years, an updated systematic review is needed to obtain a more definitive conclusion.

Therefore, the objective of this study was to assess the therapeutic efficacy of BTX-A for managing myofascial pain related to TMDs through a systematic review and meta-analysis of the literature.

## Materials and methods

This work has been registered on the Research Registry (UIN: reviewregistry1804, https://www.researchregistry.com/browse-the-registry#registryofsystematicreviewsmeta-analyses/registryofsystematicreviewsmeta-analysesdetails/65efca286a817200280df01a/). This study was conducted according to the Preferred Reporting Items for Systematic Reviews and Meta-Analyses (PRISMA, Supplemental Digital Content 1, http://links.lww.com/MS9/A484) statement guidelines^14^ (www. prisma-statement.org) (for details, see Supplemental Table 1, Supplemental Digital Content 2, http://links.lww.com/MS9/A485) alongside the AMSTAR 2 guidelines (for details, see Supplemental Digital Content 2, Supplemental Digital Content 3, http://links.lww.com/MS9/A486)^15^.

### Search strategy

The literature search was performed on August 22, 2023, and last updated on January 22, 2024. The following electronic databases were screened: PubMed, Web of Science, and the Cochrane Library. The search strategy aimed to identify all relevant articles published in English with no time restriction (for details, see Supplemental Table 1, Supplemental Digital Content 2, http://links.lww.com/MS9/A485). A supplemental manual search was conducted by reviewing the reference lists of the retrieved papers and review articles.

### Study eligibility

Two calibrated reviewers (M..Z. and Z.H.) screened the titles and abstracts (when available) of the identified studies and extracted the data in duplicate. Any disagreements between the researchers were resolved via discussion or consultation with a third reviewer (Y.W.) until a consensus was reached. Irrelevant records (bibliographic reviews, descriptive studies, animal studies, case reports, abstracts, and commentaries) were excluded, and the full texts of potentially relevant studies were obtained and reviewed. Only studies that met the following criteria were included.

#### Participants

Adult patients with TMD-related myofascial pain.

#### Intervention

BTX injection treatment.

#### Comparison

Injection of a placebo (saline solution) or underwent other specific treatments, including physical therapy, occlusal splints, drug therapy, or acupuncture.

#### Outcomes

The primary outcome was a pain score on a visual analog scale (VAS), and the secondary outcomes were maximum mouth opening (MMO) and adverse effects.

#### Studies

RCTs were also conducted in humans.

### Data extraction

Data were independently extracted by the same authors using a standardized data extraction checklist. The following data were extracted from each study and are summarized in tables: authors and publication year, diagnostic criteria and muscles involved, study design, number and average age of patients, follow-up protocol, interventions and control details, outcome variables, outcomes, and adverse effects.

### Assessment of quality and risk of bias

The risk of bias assessment was carried out using the Cochrane Risk of Bias tool (RoB 2.0; Cochrane, London, UK)^16^. Two reviewers (M.Z. and Z.H.) independently assessed the risk of bias. Discrepancies were resolved via discussion, and a third reviewer (J.Q.) was consulted if necessary. The authors of the selected studies were contacted as needed to clarify missing or unclear information.

The review authors evaluated the following domains: randomization process, deviations from intended interventions, missing outcome data, measurement of the outcome, and selection of the reported result. Each domain was assessed as having a low risk of bias, some concerns, or a high risk of bias. Then, an overall RoB judgment was assigned to each study as follows: low risk (if all domains had a low risk of bias), some concern (if at least one domain had some concerns, but none had a high risk of bias) or high risk (if one or more domains had a high risk of bias)^16^.

### Statistical analysis

The data extracted from the studies were analyzed using RevMan 5.4 software (The Nordic Cochrane Centre, The Cochrane Collaboration, Copenhagen, Denmark). Mean differences (MDs) and SDs with 95% confidence intervals (CIs) were used to summarize the data in studies with continuous outcomes. Meta-analyses were carried out to determine the effect of the BTX-A intervention on the VAS score and MMO score with respect to those of the control group. Subgroup analyses were also conducted for different follow-up times. Heterogeneity was assessed using the *I* ^2^ statistic.

## Results

### Study selection

**Figure 1:**
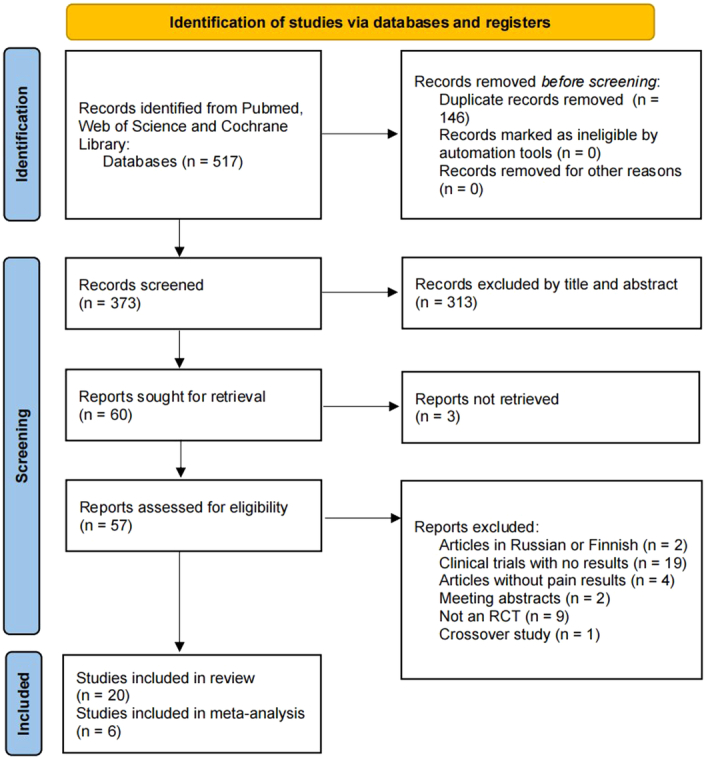
PRISMA flowchart of the search process.

A total of 373 articles were retrieved from all the databases. After screening the titles and abstracts, 313 articles that were unrelated to the topic of this systematic review were excluded. As a result, 60 articles remained for full-text assessment. Among these, 40 articles were excluded based on the predetermined eligibility criteria or because the full text could not be obtained. Finally, 20 studies were included in this systematic review and qualitative analysis^17–36^, of which six studies were included in the meta-analysis^21,24,28,31,33,35^. The flow chart of the study selection process is illustrated in Figure 1.

### Descriptions of the included studies

All 20 included studies were RCTs that compared the effect of BTX-A injection with that of a placebo or other treatment, including physical therapy, occlusal splints, drug therapy, or acupuncture, for treating TMD or TMD-related myofascial pain^17–36^. Thirteen of the 20 studies employed the research diagnostic criteria for TMD or diagnostic criteria for TMD^17–21,23–26,30–33^, while the remaining seven studies failed to specify a standardized and validated diagnostic method^22,27–29,34–36^. In 11 out of the 20 studies, BTX-A was injected into the masseter and temporal muscles^21–23,25,26,29,30,32,33,35,36^; in four studies, BTX-A was injected into the masseter, temporal, and pterygoid muscles^17,24,28,34^; in two studies, BTX-A was injected into the masseter muscle^18,31^; and in three studies, BTX-A was injected into the lateral pterygoid muscles^19,20,27^. The total dose of BTX-A ranged from 15 to 150 U for each side, and most studies used a low dose of BTX-A (30–50 U for each side). The follow-up times range from 4 to 24 weeks. Thirteen studies used saline solution as a placebo^18,19,21,23–25,28,31–36^, four studies used oral appliances or occlusal splints^20,22,25,26^, two studies used laser treatment^20,29^, and one study used fascial manipulation^30^, dry needling^27^, lidocaine injection^24^, acupuncture^23^, or percutaneous needle electrolysis^17^ as the control. Information on the diagnostic criteria of TMD, study design, number of participants, mean age or age range, follow-up period, intervention and control methods, outcome variables, and adverse effects are specified in Table 1.

**Table 1:** Main characteristics of the selected studies.

References	Diagnostic criteria and muscles involved	Study design	Number of patients	Mean age (±SD)/age range	Follow-up protocol	Intervention (*I*)	Control (C)	Outcome variables	Outcome	Adverse effect
Von Lindern *et al.* ^34^	Chronic facial pain Masseter, temporal and medial pterygoid	RCT	*I*: 60 C: 30	NR	Baseline, 4w	BTX-A: 35 U in each side of the muscle	0.9% saline solution	Pain (VAS 0–10)	Patients who received botulinum toxin improved by a significant mean reduction on subjective pain scores and there was a significant difference compared with the placebo group	Side effects in the form of swallowing difficulty or temporary paralysis of a muscle of facial expression occurred in only 1 patient and they were completely reversible after 4w
Guarda-Nardini *et al.* ^33^	RDC/TMD Masseter and temporal	RCT	*I*: 10 C: 10	25–45	Baseline, 1 w, 4w, 12w	BTX-A: 30 U in each masseter muscle and 20 U in each anterior temporal muscle	0.9% saline solution	Pain at rest and at chewing (VAS 0–10); Mastication efficiency (VAS 0–10); MMO (mm); Protrusive and laterotrusive movements (mm); Functional limitation during usual jaw movements (0–4); Subjective efficacy of the treatment (0–4); Tolerance of the treatment (0–4)	Patients treated with BTX-A had a higher subjective improvement in their perception of treatment efficacy than the placebo subjects	NR
Kurtoglu *et al.* ^32^	RDC/TMD Masseter and temporal	RCT	*I*: 12 C: 12	*I*: 29.6±12.7 (16–53) C: 23.4±4.7 (20–34)	Baseline, 2w, 4w	BTX-A: 30 U in each masseter muscle and 20 U in each anterior temporal muscle	0.9% saline solution	EMG (mV); Bio-behavioral questionnaire (pain and psychological status)	Comparisons of pain, disability, and psychological status showed no statistical difference over time for the placebo or study groups	No side effects were evident
Ernberg *et al.* ^31^	RDC/TMD Masseter	RCT	*I*: 12 C: 9	26–50	Baseline, 4w, 12w	BTX-A: 50 U in each masseter muscle	0.9% saline solution	Pain (VAS 0–100); Physical and emotional function; Global improvement; MMO; PPT and PPTol	No significant differences in pain reduction were found between BTX-A injection and saline injection in patients with persistent myofascial pain	Side effects reported by the patients the first week after injections were frequent and of varying intensity but unrelated to the drug. All side effects had resolved at the 1-month follow-up
Guarda-Nardini *et al.* ^30^	RDC/TMD Masseter and temporal	RCT	*I*: 15 C: 15	*I*: 47.7±14.3 C: 43.2±13.9	Baseline, 1h, 12w	BTX-A: 150 U for each side	Fascial manipulation	Pain (VAS 0–10); MMO	Both treatment protocols provided significant improvement over time for pain symptoms. The two treatments seem to be almost equally effective, fascial manipulation being slightly superior to reduce subjective pain perception, and botulinum toxin injections being slightly superior to increase jaw range of motion
De Carli *et al.* ^29^	Myofascial pain Masseter and temporal	RCT	*I*: 7 C: 8	Mean: 38	1d, 3d, 5d, 8d, 10d, 12d, 15d, 30d	BTX-A: 60 U in each masseter muscle and 30 U in each anterior temporal muscle; 15d later, 30 U in each masseter muscle and 15 U in each anterior temporal muscle	Low-level laser	Pain (VAS 0–10); MMO	Both therapies were effective in reducing pain, but the effect of low-level lasers was faster than the use of BTX-A. Both treatments showed no statistically significant improvement in mouth opening	NR
Gupta *et al.* ^36^	TMD Masseter and temporal	RCT	*I*: 12 C: 12	20–50	Baseline, 2w, 4w	BTX-A: 30 U in each masseter muscle and 20 U in each anterior temporal muscle	isotonic saline solution	EMG; Behavior questionnaire scores (pain and psychological status)	The behavioral questionnaire results for the study group showed a statistically significant relief from the pain. Whereas in control group, no statistically significant reduction in the pain and improvement in the daily life activities was found	No signs of any kind of adverse reaction were noted except local needle site reactions such as redness
Jadhao *et al.* ^35^	Bruxism and myofascial pain Masseter and temporal	RCT	*I*: 8 C1: 8 C2: 8	20–35	Baseline, 1w,12w, 24w	BTX-A: 30 U in each masseter muscle and 20 U in each anterior temporal muscle	C1: isotonic saline solution C2: no injections	Pain at rest and at chewing (VAS 0–5); Maximum bite force	BTX-A is effective for treatment of bruxism to reduce myofacial pain and the occlusal force compared with the placebo group	NR
Patel *et al.* ^28^	TMD Masseter, temporalis and pterygoid	RCT	*I*: 10 C: 9	NR	Baseline, 4w	IncobotulinumtoxinA: 50 U into each masseter, 25 U into each temporalis, and 10 U into each external pterygoid muscle	0.9% saline solution	Pain (VAS 0–10); Pain medication usage; Masticatory muscle tenderness	We demonstrate the utility of IncobotulinumtoxinA injection in the treatment of TMD refractory to pain medication and other conventional treatments in comparison to placebo	Patients noted no adverse events during the study
Kütük *et al.* ^27^	Myofascial pain Lateral pterygoid	RCT	*I*: 20 C: 20	*I*: 33.0±6.8 C: 34.6±9.3 (21–54)	Baseline, 6w	BTX-A: 25 U at each tigger point, 25–150 U in total	Dry needling	Pain (VAS 0–10); MMO; functional limitation (0–3); jaw strength (0–3); palpable muscular spasms (0–4)	Pain relief at rest was more effective with the use of the dry needling technique after 6w. Both treatments produced significant pain relief and improved function in patients with myofascial pain	NR
Yurttutan *et al.* ^26^	RDC/TMD Masseter and temporal	RCT	*I*: 24 C: 25	*I*: 30.5±9.95 C: 31±7.33	Baseline, 24w	BTX-A: 30 U in each masseter muscle and 15 U in each anterior temporal muscle	Occlusal splint	Pain (VAS 0–10); JFLS-8; OBC-21	Both the use of an occlusal splint and BTX injection will benefit TMD patients, and BTX therapy was more effective than the occlusal splint therapy	None of the patients reported any adverse effects related to the BTX injections or occlusal splint therapy during or after the treatment period
De La Torre Canales *et al.* ^25^	RDC/TMD Masseter and temporal	RCT	*I*1: 20 *I*2: 20 *I*3: 20 C1: 20 C2: 20	36.8± 5.6	Baseline, 1w, 2w, 3w, 4w, 12w, 24w	*I*1: BTX-A low (10 U in each temporalis and 30 U in each masseter) *I*2: BTX-A medium (20 U in each temporalis and 50 U in each masseter) *I*3: BTX-A high (25 U in each temporalis and 75 U in each masseter)	C1: 0.9% saline solution (0.4 ml in temporalis and 0.6 ml in masseter) C2: OA	Pain (VAS 0–10); PPT; EMG, Masticatory Performance, Muscle thickness, CBCT	Compared to the placebo, subjective pain of BTX-A groups was significantly lower after 14 days and up to the end of the study; however, compared with OA, no statistical differences were found. Regardless of the dose, BoNT-A was as effective as OA on MFP	A transient decline in masticatory performance and muscle contraction, and a decrease in muscle thickness and coronoid and condylar process bone volume were found as dose-related adverse effects of BoNT-A
Montes-Carmona *et al.* ^24^	RDC/TMD and DC/TMD Masseter, temporal and lateral pterygoid	RCT	*I*: 20 C1: 20 C2: 20	*I*: 42.40±5.19 C1: 42.95±7.01 C2: 45.40±6.76	Baseline, 1w, 2w, 4w, 8w, 12w, 24w	BTX-A: 12 U in each masseter muscle, 12 U in each anterior temporal muscle, 4 U in lateral pterygoid muscle, and 4 U in medial pterygoid muscle	C1: 0.9% saline solution C2: 2% lidocaine with vasoconstrictor: 0.6 ml in each masseter muscle, 0.6 ml in each anterior temporal muscle, 0.2 ml in lateral pterygoid muscle and 0.2 ml in medial pterygoid muscle	Pain (VAS 0–10); parameters of jaw range (MMO, protrusion, right and left laterotrusion); TMJ affectation questionnaires	BTX-A significantly reduced pain compared to saline and lidocaine. The effects lasted up to 6 months and were more intense in patients with localized myofascial pain than in patients with referred remote pain	No significant adverse reactions were observed
De La Torre Canales *et al.* ^23^	RDC/TMD Masseter and temporal	RCT	*I*: 18 C1: 18 C2: 18	*I*: 34.6±6.5 C1: 30.8±6.9 C2: 30.3±6.9	Baseline, 4w	BTX-A: 30 U in each masseter and 10 U in each anterior temporal muscle	C1: 0.9% saline solution C2: acupuncture	Pain (VAS 0–100), PPT, EMG	After 1 month of follow-up, all therapies reduced the self-perceived pain in patients with MFP. BTX-A was not superior to acupuncture in pain reduction, but both were superior to SS; moreover, BTX-A was the only treatment able to improve PPT values	Only patients treated with BTX-A reduced the EMG activity in the injected muscles which should be considered as an adverse effect. Besides, patients receiving BTX-A injections also reported adverse effects like edema and pain during injection, being the last also reported by the SS group
Kaya *et al.* ^22^	Bruxism and myofascial pain Masseter and temporal	RCT	*I*: 20 C: 20	Mean: 26.333 (18–45)	Baseline, 2w, 6w, 12w, 24w	BTX-A: 24 U in each side of the masseter muscle	Occlusal splint	Pain (VAS 0–10); maximum bite force	Low dose BTX-A and occlusal splint use were effective in eliminating bruxism-related pain but not superior to each other.	NR
De La Torre Canales *et al.* ^21^	RDC/TMD and DC/TMD Masseter and temporal	RCT	*I*1: 20 I2: 20 I3: 20 C: 20	18–45	Baseline, 4w, 24w	I1: BTX-A low (10 U in each temporalis and 30 U in each masseter) I2: BTX-A medium (20 U in each temporalis and 50 U in each masseter) I3: BTX-A high (25 U in each temporalis and 75 U in each masseter)	0.9% saline solution	Mandibular motion (pain-free opening, maximum unassisted and assisted opening, and right and left lateral movements), Muscle pain while palpation (0–3)	BTX-A, independent of dosage, improves mandibular range of motion and muscle pain to palpation of the masseter and temporal muscles in persistent MFP patients compared with saline injections	NR
Rady *et al.* ^20^. 2022	DC/TMD Lateral Pterygoid Muscle	RCT	*I*: 9 C1: 9 C2:9	*I*: 23.22±2.1 C1: 24.22±2.9 C2: 23.22±2.1	Baseline, 12w	BTX-A: 30 U in the lateral pterygoid muscle	C1: ARA C2: LLLT	Pain (VAS 0–10), articular disc position, joint space index, time of recovery	BTX-A and LLLT could be considered effective alternative treatment modalities to ARA regarding reducing joint pain, clicking, and improving disc position in patients with symptomatic DDwR	Patients receiving BTX-A showed diminished contra-lateral mandibular movements after injection, with no other side effects noted
Rezazadeh *et al.* ^19^	RDC/TMD Lateral Pterygoid Muscle	RCT	*I*: 18 C: 18	*I*: 28.28±7.9 C: 24.78±4.5	Baseline, 1w, 4w, 12w	BTX-A: 15 U in the lateral pterygoid muscle	Saline solution	Pain (VAS), jaw movements (MMO, lateral and protrusion movement), click severity, Helkimo index	Click and VAS decreased after BTX injection, but the difference was not statistically significant compared to the control group	NR
Ayala *et al.* ^18^	DC/TMD Masseter	RCT	*I*: 7 C: 7	Mean 29.7±5.4	Baseline, 4w	BTX-A: 30 U in the masseter	Saline solution	Pain (VAS), condyle-fossa relationship	Both BTX-A and saline injections produced a significant decrease in VAS scores, but there were no significant differences between the two groups	NR
Gonzalez-Perez *et al.* ^17^	DC/TMD Masseter, temporal and lateral pterygoid	RCT	*I*: 26 C: 26	*I*: 39.11±9 C: 41.96±9.88	Baseline, 4w, 8w, 12w	BTX-A: 16 U in each masseter muscle, 16 U in each anterior temporal muscle and 16 U in each lateral pterygoid muscle	PNE	Pain at rest and during chewing (VAS 0–10); mouth opening, lateral movements, protrusion; TMJ involvement (0–100); TMJ impairment (0–100)	Both BTA and PNE showed high efficacy and safety in reducing pain and improving muscle function for the treatment of chronic masticatory myalgia. This improvement was sustained over a 3-month period in both groups	No side effects were detected with BTA, while four cases of self-limited pain and bruising at the puncture site were reported in the PNE group

ARA, anterior repositioning appliance; BTX-A, botulinum toxin type A; C, control group; CBCT, cone beam computed tomography; d, days; DC/TMD, diagnostic criteria for temporomandibular disorders; DDwR, disc displacement with reduction; EMG, electromyography; h, hours; I, intervention group; JFLS-8, jaw function limitation scale; LLLT, low-level laser therapy; m, months; MFP, myofascial pain; MMO, maximum mouth opening; NR, not reported; OA, oral appliance; OBC-21, Oral Behavior Checklist; PNE, percutaneous needle electrolysis; PPT, pressure pain threshold; PPTol, pressure pain tolerance; RCT, randomized controlled trial; RDC/TMD, research diagnostic criteria for temporomandibular disorders; VAS, visual analog scale; w, weeks.

### Primary study outcome

The primary outcome was a pain score on the VAS. All the studies included in the present systematic review evaluated pain intensity before and after BTX-A injection or control treatment. Two studies reported a significant decrease in subjective pain or muscle pain throughout the experiment in three groups that received three different doses of BTX-A^21,25^, while no significant differences were noted among the BTX-A groups. Therefore, the subsequent meta-analysis was conducted irrespective of the dose of BTX-A administered.

Thirteen studies compared BTX-A with saline injection^18,19,21,23–25,28,31–36^. Nine of these studies showed that BTX-A injections were more effective at reducing subjective pain than saline injections^21,23–25,28,33–36^, while in the other four studies^18,19,31,32^, there were no significant differences in pain reduction between the two treatments. One of the studies also assessed the effect of lidocaine injection, but the results were not significantly different from those of saline injection^24^. Another study also used acupuncture as a positive control and showed that it was superior to saline injection for pain reduction, but the effect was not significantly different from that observed in the BTX-A group^23^.

Four studies compared BTX-A with an oral appliance^20,22,25,26^, and they all showed that both treatments were equally effective at treating persistent myofascial pain. Only one study demonstrated that BTX therapy was more effective than occlusal splint therapy^26^, and the remaining three studies showed that there was no significant difference between BTX-A and oral devices^20,22,25^.

Two studies compared low-level laser therapy with BTX-A injections^20,29^. Their results showed that both treatments were effective at reducing pain, and there were no statistically significant differences between the two treatments with respect to pain at the 1-month or 3-month follow-up visit. However, regarding the time of recovery, the two studies showed opposite results. De Carli *et al.* ^29^ showed that the effects of low-level laser therapy appeared faster than those of BTX-A injections (reductions observed on day 12 vs. day 30, respectively). In contrast, Rady *et al.* ^20^ showed that the BTX-A injection group (6.11 days) had a slightly shorter recovery time than the low-level laser group (8.89 days), but the difference was not significant.

One of the included studies compared BTX-A with the fascia manipulation technique^30^. Both treatment protocols significantly improved pain symptoms during the 3-month follow-up, and they seemed to be almost equally effective. Fascial manipulation was reported to be slightly superior in reducing subjective pain perception, while BTX injections were slightly superior in increasing the jaw range of motion. Another study compared BTX-A injection with the dry needling technique^27^. Both treatments significantly relieved myofascial pain and improved jaw range of motion during the 6 weeks of follow-up. Another study compared BTX-A injection with percutaneous needle electrolysis^17^. Both treatments showed high efficacy and safety in reducing pain and improving muscle function in patients with chronic masticatory myalgia.

### Secondary study outcomes

Regarding mouth opening, we found that in most studies, the maximum opening did not significantly change in the BTX-A group during treatment, nor was there a significant difference between the BTX-A group and the control group.

Regarding adverse effects, seven studies did not observe adverse reactions related to BTX injections during the study period^17,24,26,28,31,32,36^, whereas in three studies, adverse effects such as swallowing difficulty, temporary paralysis of the muscles responsible for facial expression, reduced electromyography activity and diminished contralateral mandibular movements were reported^20,23,34^. Another study, which used three different doses of BTX-A, also reported a reduction in muscle contraction and a decrease in muscle thickness and bone volume in the condyloid and coronoid processes as dose-related adverse effects of BoNT-A (which appears more frequently in patients receiving higher doses)^25^. The remaining nine studies lacked information concerning adverse events^18,19,21,22,27,29,30,33,35^.

### Quantitative analysis

For studies using the same control (saline solution injection), assessment methods, and follow-up periods, a meta-analysis was performed. The VAS was used to evaluate pain severity in all of these studies. Three studies used a 10-point scale^24,28,33^, with 0 indicating no pain and 10 indicating unbearable pain. One study^35^ used a five-point scale, and another study used a 100-point scale^31^. Microsoft Excel was used to adjust all the scores to a 10-point scale. The available data were segregated based on different follow-up periods for the meta-analysis.

**Figure 2:**
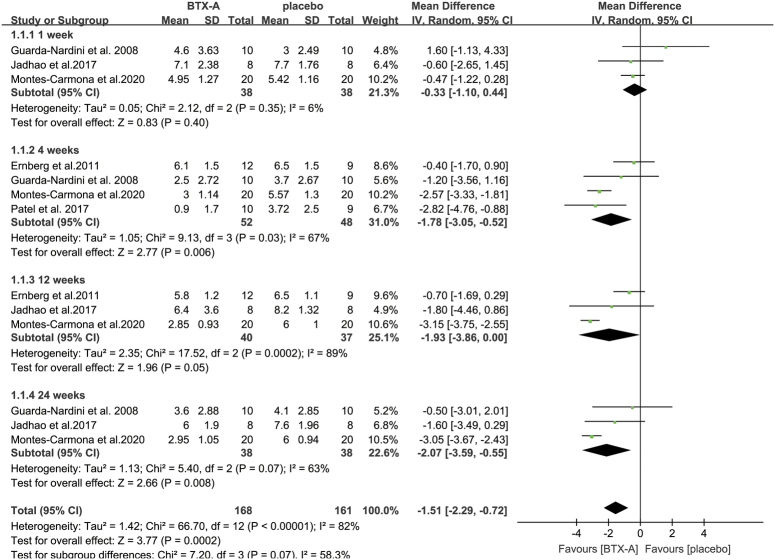
Meta-analysis of pain scores for patients receiving BTX-A injection versus placebo injection at different follow-up periods. BTX-A, botulinum toxin type A.

**Figure 3:**
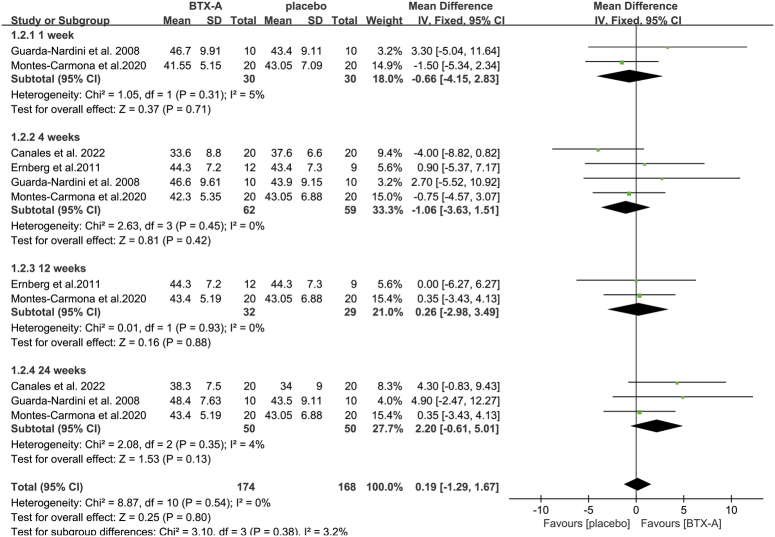
Meta-analysis of MMO with BTX-A injection versus placebo injection at different follow-up periods. BTX-A, botulinum toxin type A; MMO, maximum mouth opening.

For pain, our analysis indicated significantly lower VAS scores in patients receiving BTX-A injections than in those receiving saline solution at 4 weeks (MD: −1.78; 95% CI: −3.05, −0.52; *I* ^2^=67%; *P*=0.006), 12 weeks (MD: −1.93; 95% CI: −3.86, 0.00; *I* ^2^=89%; *P*=0.05), and 24 weeks (MD: −2.07; 95% CI: −3.59, −0.55; *I* ^2^=63%; *P*=0.008). However, there was no difference in pain scores between the two treatment modalities at 1 week (MD: −0.33; 95% CI: −1.1, 0.44; *I* ^2^=6%; *P*=0.4) (Fig. 2). Our analysis also revealed that the MMO of patients who received BTX-A did not significantly differ from that of patients who received saline at any of the time points (1 week: MD: −0.66; 95% CI: −4.15, 2.83; *I* ^2^=5%; *P*=0.71; 4 weeks: MD: −1.06; 95% CI: −3.63, 1.51; *I* ^2^=0%; *P*=0.42; 12 weeks: MD: 0.26; 95% CI: −2.98, 3.49; *I* ^2^=0%; *P*=0.88; 24 weeks: MD: 2.20; 95% CI: −0.61, 5.01; *I* ^2^=4%; *P*=0.13) (Fig. 3).

### Risk of bias analysis

**Figure 4:**
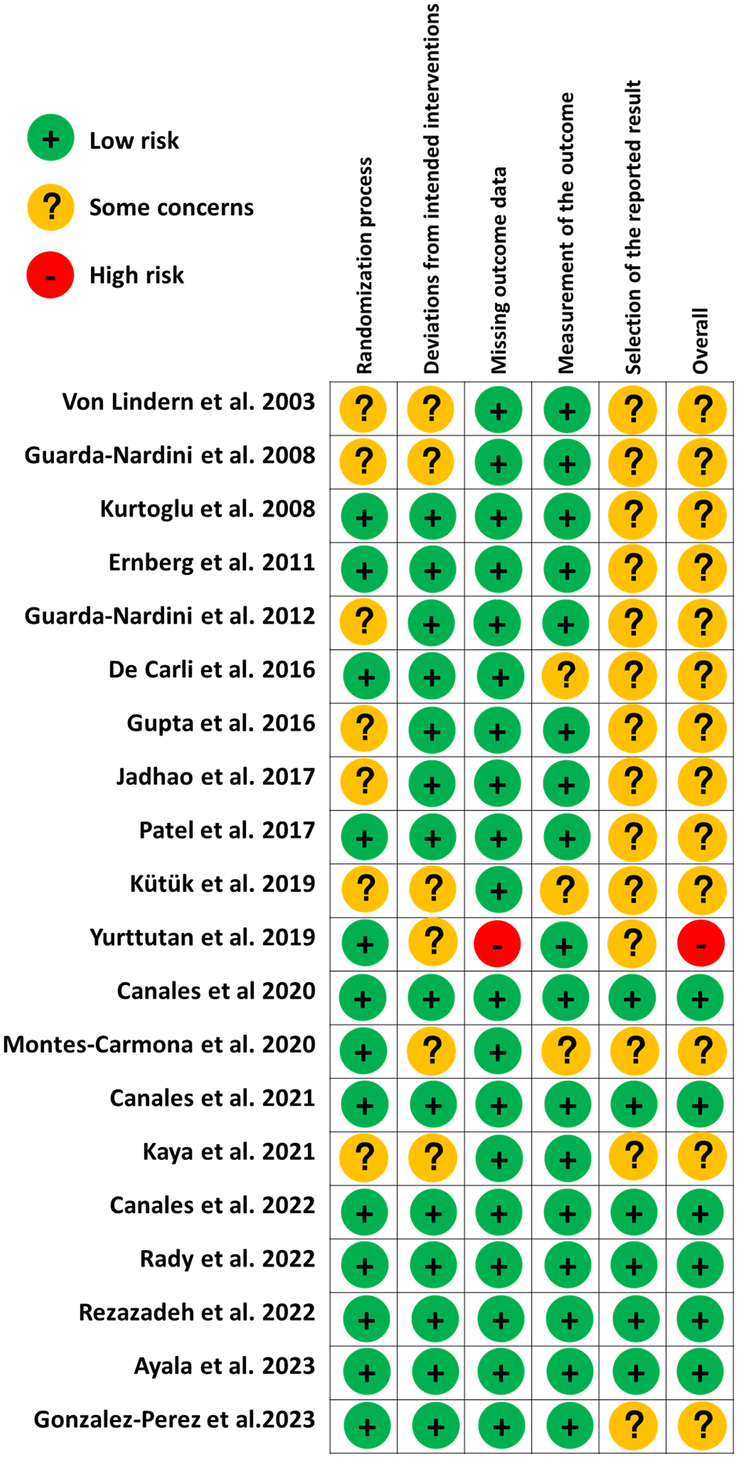
Evaluation of the included studies in terms of risk of bias.

The risk of bias assessment of the included RCTs was carried out using the Cochrane RoB 2.0 tool. Six studies had a low risk of bias^18–21,23,25^, one had a high risk of bias^26^, and 13 studies were classified as having some concerns regarding the reported results^17,22,24,27–36^. The details are presented in Figure 4.

## Discussion

TMDs accompanied by myofascial pain cause significant disruption in an individual’s psychosocial functioning and significantly alter their quality of life. Various conventional treatment methods have been reported to be effective for the treatment of TMD^37^. Nevertheless, some patients do not experience complete pain relief as a result of conventional treatment approaches. Under these conditions, intramuscular injections of BTX-A have been proposed in the literature as an alternative treatment due to its ability to relax muscles and relieve pain^9^. However, the efficacy of BTX-A treatment has not been fully elucidated because of a lack of evidence. Therefore, this systematic review aimed to assess the effects of BTX-A in patients with TMD-related myofascial pain. Our results showed that, compared with placebo treatment, BTX-A injection was more effective at reducing myofascial pain in TMD patients, and its effect was similar to that of conventional methods. However, BTX-A was demonstrated to have no significant effect on maximum opening.

In the present systematic review, BTX-A injections were demonstrated to be effective at reducing myofascial pain according to studies comparing BTX-A with a placebo (saline). Our quantitative analysis showed that BTX-A injection was superior to placebo at the 4-week follow-up visit and those thereafter but not at the 1-week follow-up visit. These results indicate that BTX-A might be effective at reducing TMD-related myofascial pain, especially after a relatively long follow-up period of more than 4 weeks. However, all the studies included in the meta-analysis were classified as having an unclear risk of bias. Therefore, conclusions should still be drawn with caution. On the other hand, there was no difference in MMO between the two groups at any time point, which is consistent with the findings of a recent network meta-analysis^38^. This result suggested that for TMD patients with the chief complaint of restricted mouth opening, treatment with BTX-A alone might not yield satisfactory results.

Due to its noninvasiveness and reversibility an oral appliance is likely the most widely used therapy for reducing the symptoms of myofascial pain^25^. According to our systematic review, patients treated with BTX-A had equivalent or better outcomes than patients treated with oral appliances. However, strict compliance with the use of oral appliances limits their use, and their effectiveness is unguaranteed. Therefore, for patients who are not eligible for treatment with a removable oral appliance or who have poor compliance, BTX-A injection can be considered an alternative.

Other conventional treatments (fascial manipulation technique, low-level laser therapy, dry needling technique, and percutaneous needle electrolysis) presented similar effects as BTX-A in terms of pain reduction. However, the number of studies was very limited. In the future, additional clinical studies comparing the effects of BTX-A with those of conventional treatments should be conducted before any definitive recommendations can be made. Currently, the use of BTX-A for myofascial pain treatment could be considered for patients for whom conservative management has failed.

Two studies demonstrated that different doses of BTX-A were equally effective at treating myofascial pain related to TMD^21,25^. However, patients in the groups who received medium (70 U on each side) and high (100 U on each side) doses of BTX-A were reported to experience adverse effects, such as reduced muscle activity and decreased muscle thickness, which were not observed in the group who received low doses (40 U on each side) of BTX-A. Therefore, BTX-A should ideally be administered at low doses in patients with myofascial pain to minimize possible adverse effects. Besides the two studies mentioned above, another eight studies included also used a low dose of BTX-A (30–50 U on each side) and showed that BTX-A is more effective for reducing myofacial pain compared with the control group^23,24,26,28,33–36^. However, two recent studies using a lower dose (30 U in the masseter or 15 U in the lateral pterygoid muscle, respectively) of BTX-A reported no significant differences in pain reduction between patients who received BTX-A and those who received a placebo injection^18,19^. Therefore, the ideal dose of BTX-A for treating myofascial pain in TMD patients remains to be determined.

Adverse effects should not be ignored when BTX-A treatment is considered. In their meta-analysis and systemic review, Naumann and Jankovic^39^ reported no severe adverse events, with focal weakness as the only complaint. In our review, a few studies reported adverse effects related to BTX-A, such as swallowing difficulty, temporary paralysis of the muscles responsible for facial expression, reduced electromyography activity, and diminished contralateral mandibular movements. Therefore, the tradeoff between effectiveness and the probability of developing adverse effects should be assessed carefully when considering BTX-A as an option for TMD-related myofascial pain. Future studies should also assess the duration of adverse effects and whether multiple applications of low doses of BTX-A could lead to the development of adverse effects.

Finally, this systematic review has several limitations. First, there may be selection bias because the search was restricted to publications written in English. Additionally, all the comparisons that were performed included a small number of studies, which may have contributed to the low power of the meta-analyses. Another limitation is the heterogeneity observed in some of the analyzed outcomes, such as visual analog pain score at 4, 12, and 24 weeks. The heterogeneity observed may stem from the variations in the BTX-A injection doses and sites of the included studies. Therefore, additional RCTs with larger sample sizes and longer follow-up periods with low risk of bias and lower heterogeneity are needed to determine the effectiveness of BTX-A for the long-term treatment of myofascial pain in patients with TMD.

## Conclusions

Within the limitations of this systematic review, the evidence suggests that BTX-A is effective at relieving pain in TMD patients but not at improving mouth opening. Furthermore, due to the adverse effects mentioned above and the invasiveness of the injection, conservative treatments should remain the first-line remedy for TMD-associated myofascial pain. Nevertheless, we suggest administering a low dose of BTX-A to TMD patients who do not experience complete pain relief as a result of conservative treatments but not to those whose mouth opening is restricted. In the future, additional high-quality, well-designed clinical studies on this topic should be conducted before any definitive recommendations can be made.

## Ethics approval

Not applicable.

## Consent

Not applicable.

## Sources of funding

This work was supported by grants from the Project of Shanghai Municipal Health Commission (grant no. 20214Y0080), Xuhui Municipal Health Commission (grant no. SHXH202210), and the Medical Key Subject of Xuhui District (grant no. SHXHZDXK202302).

## Author contribution

M.Z. and Z.H.: contributed to the conception, design, data acquisition, analysis, and interpretation, drafted and critically revised the manuscript. Y.W. and J.Q.: contributed to data acquisition and analysis and critically revised the manuscript. M.F.: contributed to the conception, design, data acquisition, analysis, and interpretation, critically revising the manuscript. All authors gave final approval and agreed to be responsible for all aspects of the work.

## Conflicts of interest disclosure

There are no conflicts of interest.

## Research registration unique identifying number (UIN)

Registered with research registry (UIN: reviewregistry1804).

## Guarantor

Mingyue Fan.

## Data availability statement

The datasets used and/or analyzed during the current study are available from the corresponding author upon reasonable request.

## Provenance and Peer review

Not commissioned, externally peer-reviewed.

## Supplementary Material

SUPPLEMENTARY MATERIAL
