# Supplementary material for: Effects of botulinum toxin type A in patients with painful temporomandibular joint disorders: a systematic review and meta-analysis
Source: Ann Med Surg (Lond). 2024 May 20;86(7):4112–22. doi: 10.1097/MS9.0000000000002183 (PMC11230827; doi:10.1097/MS9.0000000000002183)
Supplement: SUPPLEMENTARY MATERIAL [file ms9-86-4112-s002.docx]

**Supplemental table 1. Search strategy used for each database**

**Table A.** Search strategy for PubMed

| #1 | “Temporomandibular joint dysfunction syndrome” OR “Temporomandibular joint disorders” [MeSH] |
| --- | --- |
| #2 | “Temporomandibular joint disorder” OR “Temporomandibular disorder” OR “Temporomandibular joint dysfunction syndrome” OR “TMD” [Title/Abstract] |
| #3 | “Temporomandibular joint” OR “TMJ” [Title/Abstract] |
| #4 | #1 OR #2 OR #3 |
| #5 | “botulinum toxins, type A” OR “botulinum toxins” [MeSH] |
| #6 | “botulinum toxin” OR “botulinum” [Title/Abstract] |
| #7 | “botulinum toxin type A” OR “BTX-A” OR “BoNT-A” [Title/Abstract] |
| #8 | “incobotulinumtoxinA” OR “inco-BoNT/A” [Title/Abstract] |
| #9 | “bocouture” OR “xeomeen” OR “botox” OR “xeomin” [Title/Abstract] |
| #10 | #5 OR #6 OR #7 OR #8 OR #9 |
| #11 | #4 AND #10 |

**Table B.** Search strategy for Web of Science

| #1 | Topic: (Temporomandibular joint dysfunction syndrome) OR Topic: (Temporomandibular joint disorder) OR Topic: (Temporomandibular disorder) OR Topic: (TMD) |
| --- | --- |
| #2 | Topic: (Temporomandibular joint) OR Topic: (TMJ) |
| #3 | #1 OR #2 |
| #4 | Topic: (botulinum) OR Topic: (botulinum toxins) |
| #5 | Topic: (botulinum toxin type a) OR Topic: (BTX-A) OR Topic: (BoNT-A) |
| #6 | Topic: (incobotulinumtoxinA) OR Topic: (inco-BoNT/A) |
| #7 | Topic: (bocouture) OR Topic: (xeomeen) OR Topic: (botox) OR Topic: (xeomin) |
| #8 | #4 OR #5 OR #6 OR #7 |
| #9 | #3 AND #8 |

**Table C.** Search strategy for Cochrane Library

| #1 | MeSH descriptor: [Temporomandibular Joint Dysfunction Syndrome] explode all trees |
| --- | --- |
| #2 | [Temporomandibular Joint Disorders] explode all trees |
| #3 | (temporomandibular joint):ti,ab,kw OR (TMJ):ti,ab,kw (Word variations have been searched) |
| #4 | (Temporomandibular joint disorder)ti,ab,kw OR (Temporomandibular disorder);ti,ab,kw OR (Temporomandibular joint dysfunction syndrome ):ti,ab,kw OR(TMD):ti,ab,kw (Word variations have been searched) |
| #5 | #1 OR #2 OR #3 OR #4 |
| #6 | MeSH descriptor: [Botulinum Toxins, Type A] explode all trees |
| #7 | MeSH descriptor: [Botulinum Toxins] explode all trees |
| #8 | (botulinum toxins):ti,ab,kw OR (botulinum):ti,ab,kw OR (botulinum toxin, type a):ti,ab,kw (Word variations have been searched) |
| #9 | (BTX-A):ti,ab,kw OR (BONT-A):ti,ab,kw OR (incobotulinumtoxinA):ti,ab,kw OR (inco-BoNT-A) |
| #10 | (bocouture):ti,ab,kw OR (xeomeen):ti,ab,kw OR (botox):ti,ab,kw OR (xeomin):ti,ab,kw |
| #11 | #6 OR #7 OR #8 OR #9 OR #10 |
| #12 | #5 AND #11 |
